# Proteomic Analyses of Nucleoid-Associated Proteins in *Escherichia coli, Pseudomonas aeruginosa, Bacillus subtilis,* and *Staphylococcus aureus*

**DOI:** 10.1371/journal.pone.0019172

**Published:** 2011-04-26

**Authors:** Ryosuke L. Ohniwa, Yuri Ushijima, Shinji Saito, Kazuya Morikawa

**Affiliations:** Institute of Basic Medical Sciences, Graduate School of Comprehensive Human Sciences, University of Tsukuba, Tennodai, Tsukuba, Ibaraki, Japan; St. Georges University of London, United Kingdom

## Abstract

**Background:**

The bacterial nucleoid contains several hundred kinds of nucleoid-associated proteins (NAPs), which play critical roles in genome functions such as transcription and replication. Several NAPs, such as Hu and H-NS in *Escherichia coli*, have so far been identified.

**Methodology/Principal Findings:**

Log- and stationary-phase cells of *E. coli*, *Pseudomonas aeruginosa*, *Bacillus subtilis*, and *Staphylococcus aureus* were lysed in spermidine solutions. Nucleoids were collected by sucrose gradient centrifugation, and their protein constituents analyzed by liquid chromatography-mass spectrometry/mass spectrometry (LC-MS/MS). Over 200 proteins were identified in each species. Envelope and soluble protein fractions were also identified. By using these data sets, we obtained lists of contaminant-subtracted proteins enriched in the nucleoid fractions (csNAP lists). The lists do not cover all of the NAPs, but included Hu regardless of the growth phases and species. In addition, the csNAP lists of each species suggested that the bacterial nucleoid is equipped with the species-specific set of global regulators, oxidation-reduction enzymes, and fatty acid synthases. This implies bacteria individually developed nucleoid associated proteins toward obtaining similar characteristics.

**Conclusions/Significance:**

Ours is the first study to reveal hundreds of NAPs in the bacterial nucleoid, and the obtained data set enabled us to overview some important features of the nucleoid. Several implications obtained from the present proteomic study may make it a landmark for the future functional and evolutionary study of the bacterial nucleoid.

## Introduction

The genomes of all living organisms are packed in cells with proteins involved in various cellular processes such as transcription and replication. Most bacteria have circular genomes of various sizes (*Staphylococcus aureus*: 1 mm; *Bacillus subtilis*: 1.4 mm: *Escherichia coli*: 1.6 mm; *Pseudomonas aeruginosa*: 2.1 mm; per genome [estimated according to a base pair size of 0.34 nm]), which are packed into cells of a few micrometers in the form of a “nucleoid” [1], [2].

Over 300 protein species are expected to be associated with the nucleoids isolated from *E. coli* and *B. subtilis*, although most of them have yet to be identified [3]–[6]. In the case of *E. coli*, several proteins have been identified as major nucleoid-associated proteins (NAPs): heat-unstable nucleoid protein (HU), integration host factor (IHF) (Hu paralogue), histone-like nucleoid structuring protein (H-NS), factor for inversion stimulation (Fis), host factor for RNA phage Qβ replication (Hfq), suppressor of T4 *td* mutant phenotype A (StpA) (H-NS homologue), and DNA-binding protein from starved cells (Dps) [7], [8]. These proteins occupy wide portions of genomic DNA [9], [10] and are involved in a series of genome functions, such as transcription (Hu, IHF, H-NS, StpA, and Fis) [11]–[15], translation (Hu, HNS, StpA, and Hfq) [16]–[19], replication (HU, IHF, Fis, and Dps) [20]–[23], DNA protection (Dps) [24], [25], and DNA packing (Hu, H-NS, Fis, and Dps) [26]–[30].

These NAPs are not quantitatively static throughout growth [31]. Hu and Fis are abundant in the log phase but decrease toward the stationary phase. Conversely, IHF and Dps are induced toward the stationary phase and become the major components. Under anaerobic conditions, DNA-binding protein in anaerobic conditions (DAN) becomes the most abundant NAP [32]. In addition, comparative genomic analysis revealed that these *E. coli* NAPs are not commonly conserved in the bacterial kingdom [33], [34] (see Table 1). Fis, H-NS, and StpA are present only in the Gammaproteobacteria. About half of all bacteria lack Hfq. Chlamydia and some of the Proteobacteria, Actinobacteria, and Firmicutes lack Dps. Even the most broadly conserved NAP, Hu or its homologue IHF, is absent in *Leptospira interrogans* and *Corynebacterium diphtheriae*. Thus, the features of the major NAPs of *E. coli* suggest the necessity of a comparative study of NAPs to gain insight into the general/specific characteristics of the bacterial nucleoid.

**Table 1:** Major NAPs Identified in This Study.

			*hupA*	*hupB*	*ihfA*	*ihfB*	*hns*	*stpA*	*fis*	*hfq*	*dps*
log phase	*E. coli*	Protein amount*	55,000		10,000		20,000	25,000	60,000	57,000	7,500
	*E. coli*	Nucleoid fraction+	47.63	3.3	0.35	-	1.45	3.89	0.36	0.84	0.46
		Envelop fraction+	-	-	-	-	-	-	0.36	-	0.21
		Top fraction+	-	-	-	-	0.56	-	-	0.84	0.46
	*P. aeruginosa*	Nucleoid fraction+	0.42	1.09	-	-	×	×	0.34	1.09	-
		Envelop fraction+	-	-	-	-	×	×	-	-	-
		Top fraction+	-	-	-	-	×	×	-	-	-
	*B. subtilis*	Nucleoid fraction+	-	0.98	×	×	×	×	×	×	-
		Envelop fraction+	-	-	×	×	×	×	×	×	-
		Top fraction+	-	-	×	×	×	×	×	×	-
	*S. aureus*	Nucleoid fraction+	-	0.42	×	×	×	×	×	×	-
		Envelop fraction+	-	-	×	×	×	×	×	×	-
		Top fraction+	-	-	×	×	×	×	×	×	-
stationary phase	*E. coli*	Protein amount*	15,000		28,000		6,500	9,000	0	17,500	160,000
	*E. coli*	Nucleoid fraction+	0.42	-	0.35	0.89	0.96	0.25	-	-	0.46
		Envelop fraction+	-	-	-	-	-	-	-	-	0.21
		Top fraction+	-	-	-	-	-	-	-	-	0.76
	*S. aureus*	Nucleoid fraction+	-	1.85	×	×	×	×	×	×	-
		Envelop fraction+	-	-	×	×	×	×	×	×	-
		Top fraction+	-	-	×	×	×	×	×	×	-

*The amount of molecules were determined according to Azam et al, 1999 (molecules/cell).

+emPAI values.

- Not detected; × Absence of gene.

The biochemical methods for purifying the cell membrane and cell wall fractions have been established [35], [36], and the total proteins in such purified fractions have been identified by mass spectrometry techniques (reviewed in [37]). However, difficulty in isolating nucleoids remains. The isolated nucleoid under physiological salt conditions always includes contaminants derived from the cell membrane, cell wall, and cytosol [4], [5], [38]–[43]. Treatment with high salt and/or RNase can disassociate the contaminated proteins, but the number of proteins identified in this manner was limited [44]–[46].

In this study, we identified the proteins in the isolated spermidine nucleoids of *E. coli*, *P. aeruginosa*, *B. subtilis*, and *S. aureus* by liquid chromatography-mass spectrometry/mass spectrometry (LC-MS/MS). Nucleoids isolated in a spermidine solution with mild ionic strength retain the most proteins directly/indirectly bound to the genomic DNA [5], [39]. The comparison of proteins detected in the nucleoid and envelope and soluble fractions suggested some characteristics of the bacterial nucleoid. We discuss several implications yielded by the obtained NAP data sets regarding the possible functional and evolutionary aspects of the bacterial nucleoid.

## Results

### Identification of NAPs in *E. coli*

**Figure 1:**
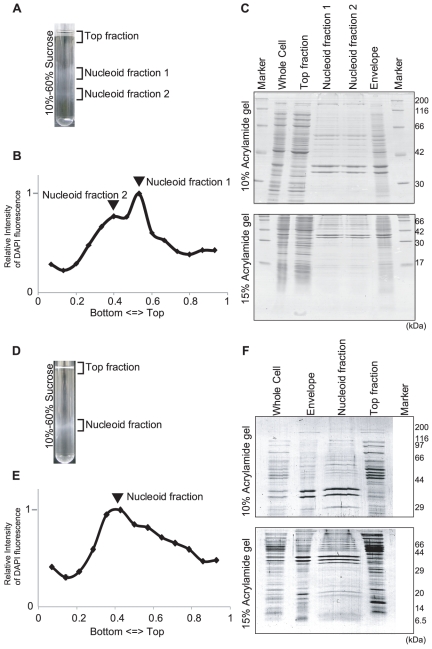
*E. coli* nucleoid isolation. Isolation of spermidine nucleoids in the log (A to C) and stationary (D to F) phases. (A) (D) The lysed cells were fractionated by sucrose-gradient centrifugation with a 10%-to-60% sucrose gradient. (B) (E) The relative DNA amount in the sucrose gradient detected by DAPI fluorescence. (C) (F) SDS-PAGE of the whole-cell lysates, the top fraction of the sucrose gradient, the nucleoid fractions, and the envelope fraction. The gels were stained with Coomassie Brilliant Blue (CBB). The pattern of the envelope in the log phase was similar to that reported by Lai et al [89].

**Figure 2:**
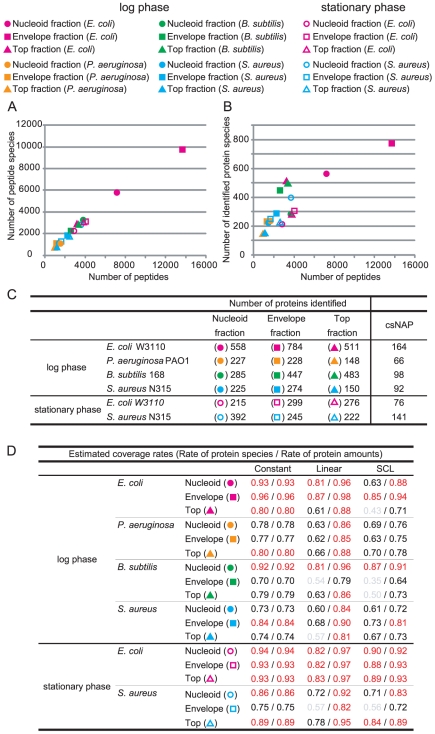
Statistical information for the identified proteins. (A, B) Relation between the number of peptide species, the total number of peptides, and the number of identified protein species in the nucleoid (circle), envelope (square), and top (triangle) fractions of *E. coli* (pink), *P. aeruginosa* (orange), *B. subtilis* (green), and *S. aureus* (blue) in the log (blank) and stationary (filled) phases (C) Number of identified protein species in each fraction csNAP represents contaminant-subtracted NAP. (D) The estimated coverage rates in each fraction. Constant, Linear, and SCL indicate the models representing the amounts of individual proteins in the fractions. The rates gaining over 0.80 are colored red, from 0.60 to 0.79, black, and less than 0.60, grey.

We isolated spermidine nucleoids from *E. coli* cells grown in aerobic conditions (see Figure S1 for growth curves and sampling points). DNA content was monitored for each sucrose gradient fraction (Figure1A, 1B, 1D and 1E), and the one with the highest DNA content was further analyzed as the nucleoid fraction. In the case of the log phase, multiple peaks were sometimes observed, probably owing to the viscous characteristics of the nucleoid (Figure 1A-1B). It might be because of the heterogeneous density of nucleoids: e.g. existence of the stationary-phase type nucleoid (Figure 1E). However, analysis by SDS-PAGE showed that the protein signal patterns of fractions 1 and 2 were indistinguishable (Figure 1C). The upper fraction (fraction 1) was analyzed by LC-MS/MS. We also analyzed proteins in the envelope and top fractions, as described in the Materials and Methods section (Figure 1C and 1F). The numbers of the identified proteins and peptides are summarized in Figure 2A to 2C. The full list of identified proteins with the number of corresponding peptides is provided in the supplementary tables (Table S1, S2, and S3).

The number of the detected peptides was correlated with the number of identified proteins (Figure 2B). This implied that the identified protein sets with lower numbers of peptides were not sufficient to cover all the proteins in the given fractions. By using *in silico* simulation, we estimated the coverage rates, which represent the extent of the identified proteins out of the total proteins in the fractions (Figure 2D, see Materials and Methods). Here, we applied 3 different models representing the amounts of individual proteins in the factions (Figure S2). The constant model represents the uniform distribution of protein amounts, and the linear model represents the linear decrease of the distribution. The simplified canonical law (SCL) model has been reported to be consistent with the relative expression of proteins in prokaryotic cells [47]. In the case of the nucleoid fractions of the log phase, the coverage rates of the identified protein species according to these models were estimated as 0.63 to 0.93, and the rates of protein amounts covered by the identified proteins as 0.88 to 0.96. In the case of the stationary phase, the rates of the species and amounts were 0.82 to 0.94 and 0.92 to 0.97, respectively. Thus, it can be expected that over 60% of the protein species that occupy at least 80% of the protein amounts out of the total proteins in the nucleoid fractions were identified in *E. coli*. The rates of the envelope and top fractions were 0.43 to 0.96 for the protein species, and 0.71 to 0.98 for the protein amounts. These data suggest that we successfully identified at least 70% of protein amounts although there may remain various unidentified protein species in each fraction. In Table S4, we summarized the potential number of protein species in each fraction and the numbers of the peptides required for identifying all of them. The number of peptides required to identify all the proteins in each fraction were estimated based on the SCL model (Table S4).

**Figure 3:**
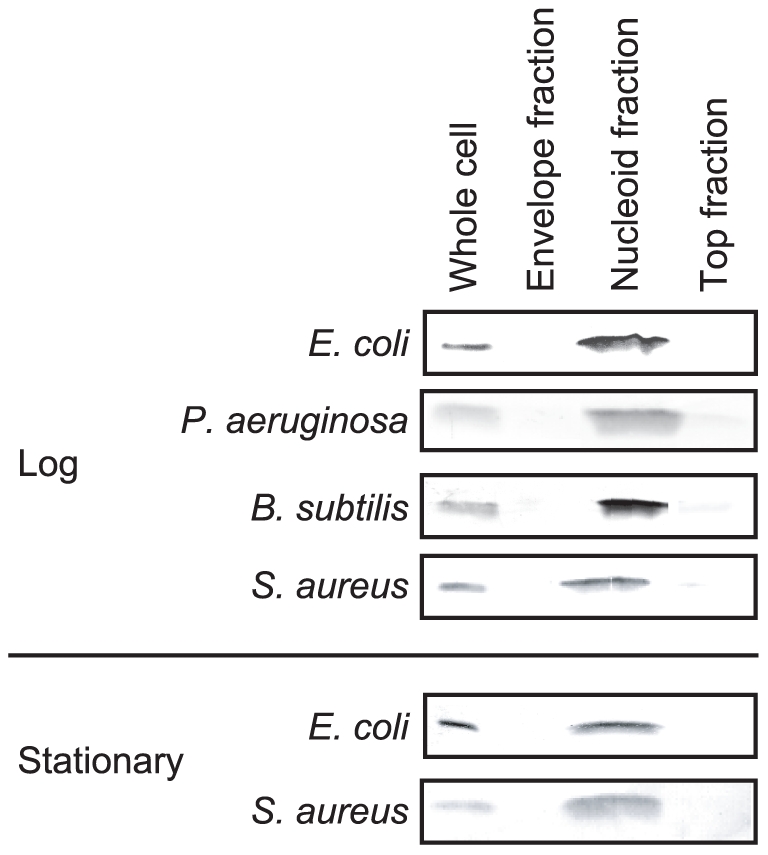
Western blots against Hu. Five micrograms of proteins from the whole-cell lysates, the nucleoid fractions, the envelope fractions, and the top fractions were separated by SDS-PAGE, and Hu was detected by Western blotting. ‘Log’ and ‘Stationary’ represent the log phase and stationary phase, respectively.

Major NAPs were identified in the nucleoid fractions (Table 1). Hu was detected in the nucleoid fraction in both the log and stationary phases, consistent with the Western blot analysis against Hu (Figure 3). In the nucleoid fraction of the log phase, Hu (*hupA* and *hupB*), IHF (*ihfA*), HNS (*hns*), StpA (*stpA*), Fis (*fis*), Hfq (*hfq*), and Dps (*dps*) were identified. In the stationary phase, Hu (*hupA*), IHF (*ihfA* and *ihfB*), HNS (*hns*), StpA (*stpA*), and Dps (*dps*) were identified. Their emPAI values, which roughly represent the amount of proteins detected by MS [48], were correlated with the intracellular amount of all of these proteins other than Fis and Hfq (Table 1).

In addition to the major NAPs, various DNA and RNA binding proteins were identified in the nucleoid fractions (Table 2). Twenty-six transcription factors were identified in the log phase, and three in the stationary phase. RNA polymerases and ribosomal proteins, which have been identified in spermidine nucleoids [5], were also identified in both phases. Other proteins related to translation (*tsf*, *infC* etc.), replication (*seqA*, *topA* etc.), and DNA repair (*mutS*, *uvrA* etc.) were also included. Non-DNA binding proteins should be included in the isolated nucleoids. For example, it is known that transcription elongation factors Rho (*rho*) and NusA (*nusA*) interact with RNA polymerase, and, indeed, our list included them.

**Table 2:** DNA and RNA Binding Proteins Identified in the Nucleoid Fractions (except major NAPs).

Category	Phase	Species	Genes
Transcription factors	log phase	*E. coli*	*arcA* *, *ascG, chbR* *, *crl* *, *crp* *, *cspE* *, *fruR* *, *fur* *, *idnR* *, *lacI* *, *lldR* *, *lrp* *, *malT* *, *osmE* *, *ompR* *, *phoU* *, *pspB* *, *purR* *, *putA* *, *rtcR* *, *srlR* *, *ybaD, yciT* *, *ydeW, ydfH* *, *yheO* *
		*P. aeruginosa*	*crp, fabG, yebK, yhbY*
		*B. subtilis*	*degA, gabR, gntR, kdgR, rsfA, spo0J, xylR, ybbB, ydiP, yhdQ, yplP*
		*S. aureus*	*codY, fabG, graR, rex, rot, sarA, sarH1, sarR, spxA, srrA, vraR*
	stationary phase	*E. coli*	*osmE* *, *lysR* *, *putA* *
		*S. aureus*	*ahrC, codY, fabG, graR, mgrA, nreC, pyrR, rocA, saeR, sarA, sarR, sarH1, sarV, sarZ, srrA, tcaR, vraR, vicR*
Proteins involved in transcription, translation, replication, and DNA repair	log phase	*E. coli*	*deaD, fusA, treA, hrpA, hsdS, infB, infC, insB, insI, mfd, mutS, nusA, nusG, parC* *, *pcnB, pnp, polA* *, *rdgC, rhlB, rho, rimM, rlmB, rmuC, rnb, rne, rpoA* *, *rpoB* *, *rpoC* *, *rpoD* *, *rusA, seqA* *, *srmB, topA* *, *tsf, tufB, unvrA* *, *xthA, yejK, ygjF*
		*P. aeruginosa*	*efp, fusA, gyrA, gyrB, infB, infC, mutS, nusG, parC, parE, pnp, rdgC, recA, rhlB, rho, rne, rpoA, rpoB, rpoC, rpoD, ssb, topA, tsf, tufA, uvrA, yejK*
		*B. subtilis*	*fusA, gidB, gyrA, gyrB, ihfB, ihfC, mutL, mutS, nusA, pnpA, polA, polC, rpoA, rpoB, rpoC, sigF, smc, tsf, tuf, uvrA, uvrC, ydbR, yhaM, yhcR, yirY, yqfR, yqjW*
		*S. aureus*	*fus, efp, tsf, tufA, end4, ermA, infA, nusG, pnpA, recA, rnc, rnh3, rpoA, rpoB, rpoC, rpoE, uvrC, xerD*
	stationary phase	*E. coli*	*hrpA, rdgC, rpoA* *, *rpoB* *, *rpoC* *, *rpoD* *, *rpoZ* *, *rusA, ruvA, topA* *, *tsf, tufB, uvrA* *, *yejK*
		*S. aureus*	*lig, dnaN, fus, efp, tsf, tufA, gyrB, hsdR, infA, infB, infC, mfd, nusG, parC, parE, pnpA, rnc, rnj1, rnj2, rpoA, rpoB, rpoC, rpoE, gidB, ruvA, ssb, topA, Y1885*

Underlined genes represent the proteins selected as contaminant-subtracted NAPs (csNAPs).

*Genes reported as DNA-binding proteins in EcoSal [1].

**Figure 4:**
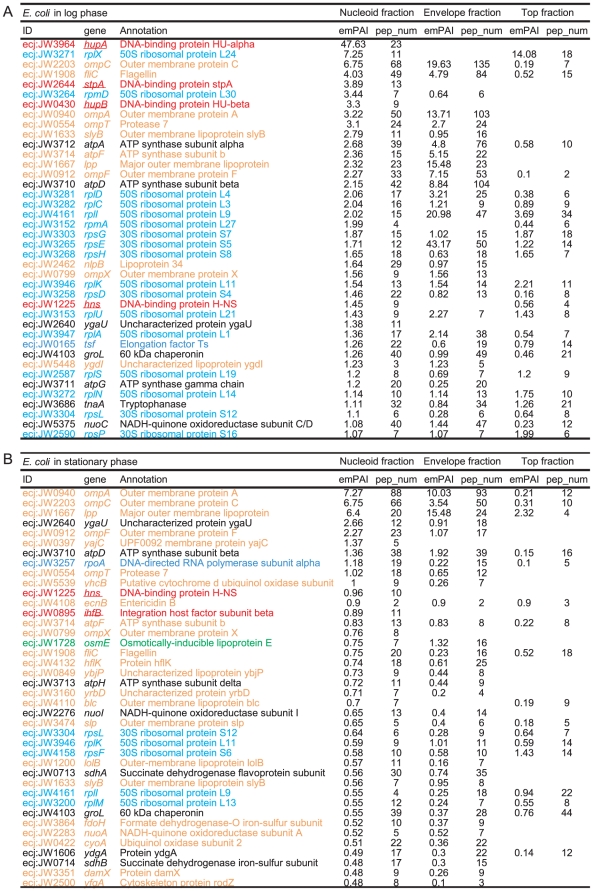
Proteins identified in the nucleoid fractions of *E. coli*. The listed proteins were the top 40 proteins sorted by the emPAI values. The colors of the letters indicate the categories of the proteins (red: major NAPs; green: transcription factors; dark blue: DNA/RNA binding proteins involved in transcription, translation, replication, and DNA repair; light blue: ribosomal proteins; black: cytosolic-type proteins; and orange: envelope-type proteins). The major NAPs, transcription factors, and DNA/RNA binding proteins were classified according to EcoSal [1] and their annotations by KEGG [86]. The ribosomal proteins were based on gene annotations. The residual proteins were classified into cytosolic-type and envelope-type proteins according to the prediction of their intracellular localization. The localizations of *E. coli* proteins were predicted by EchoLOCATION [90]. Those of *P. aeruginosa, B. subtilis, and S. aureus* were predicted by PSORTb [91]. Underlined genes were reported as DNA binding proteins in EcoSal. pep_num represents the number of peptides detected from each protein.

The nucleoid fractions were enriched in the envelope and cytosolic proteins (Figure 4, Table S2, and Table S3). When the proteins in the nucleoid fractions were sorted by emPAI value, 18 out of the top 40 proteins were envelope and cytosolic proteins in the log phase (Figure 4A). These include F_0_F_1_-ATPase (coded by *atpA*, *atpD*, etc.), porins (*ompA*, *ompC*, etc.), flagellin (*fliC,* etc.), a chaperone (*groEL*), and metabolic enzymes (*tnaA*, *nuoC*, etc.). In the stationary phase, 31 out of the 40 proteins were envelope or cytosolic proteins including F_0_F_1_-ATPase, porins, flagellin, a chaperone, and metabolic enzymes (Figure 4B). Most of these proteins were also identified in the envelope and/or top fractions with high emPAI values, suggesting that these proteins in the nucleoid fractions are contaminated largely owing to the difficulty of nucleoid isolation (see Discussion).

### NAPs in P. aeruginosa, B. subtilis, and S. aureus

We investigated the NAPs of *P. aeruginosa*, *B. subtilis*, and *S. aureus*. *P. aeruginosa* is Gram-negative and, like *E. coli,* belongs to the Gammaproteobacteria. *B. subtilis* and *S. aureus* are both Gram-positive and belong to the Firmicutes/Bacillales. The number of major *E. coli* NAP genes varies depending on the species (Table 1). *P. aeruginosa* possesses *hupA*, *hupB*, *ihfA*, *ihfB*, *fis*, *hfq*, and *dps*, but not *hns* and *stpA* (but possesses *mvaT* and *mvaU* as functional counterparts of *hns* and *stpA* [49]). *B. subtilis* possesses only *hupB* (annotated as *hbs),* its homologues, *yonN,* and *dps*. *S. aureus* possesses *hupB* (*hu*) and *dps* (*mrgA*) but lacks *fis*, *hns*, *stpA*, and *hfq*.

**Figure 5:**
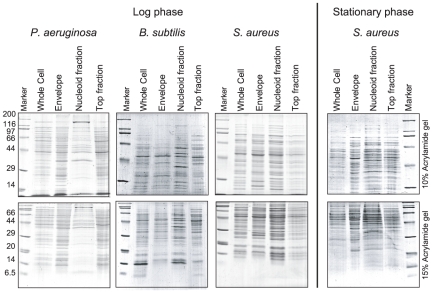
Proteins in the nucleoid, top, and envelope fractions of *S. aureus*, *P. aeruginosa*, and *B. subtilis*. SDS-PAGE analysis of the whole-cell lysates, envelope fractions, nucleoid fractions, and top fractions of the sucrose gradient assay. The gels were stained with CBB.

Proteins in the spermidine-nucleoid fractions, as well as in the envelope and top fractions, were isolated (Figure 5) and identified by LC-MS/MS (Figure 2, Figure S3, Tables S1 and S5, S6, S7, S8). Over 200 proteins were identified in each nucleoid fraction. The coverage rates of the proteins in the nucleoid fractions were 0.60 to 0.92 as protein species and 0.73 to 0.96 as protein amounts (Figure 2D), suggesting that major part, but not all, of the nucleoid proteins were identified (Table S4). Hu was exclusively detected in the nucleoid fractions of all species tested. This was consistent with the Western blots against Hu (Figure 3). In the case of *P. aeruginosa*, Hu (*hupA* and *hupB*), Fis (*fis*), and Hfq (*hfq*) were detected in the nucleoid fraction, but IHF (*ihfA* and *ihfB*), Dps (*dps*), MvaT (*mvaT*), and MvaU (*mvaU*) were not. In the case of *B. subtilis* and *S. aureus*, HU (*hbs*, *yonN*, and *hu*) was identified, but Dps (*dps* and *mrgA*) was not. DNA and RNA binding proteins other than ribosomal proteins are listed in Table 2. In all species tested, as in *E. coli*, many ribosomal proteins, envelope proteins, and cytosolic proteins were identified in the nucleoid fractions (Table S1 and Table S5, S6, S7, S8).

### Data analysis: contaminant-subtracted NAPs (csNAPs)

The isolated nucleoids contained various envelope and cytosolic proteins. This was the expected result, as described in the Introduction section. To deduce reasonable considerations in the Discussion section, we here define “contaminant-subtracted NAPs (csNAPs)” as follows:

csNAPs  =  “Proteins detected only in the nucleoid fraction” + “Proteins calculated to be relatively abundant in the nucleoid fraction.”

Proteins detected only in the nucleoid fraction”: Proteins detected only in the nucleoid fraction in a given condition.
Proteins calculated to be relatively abundant in the nucleoid fraction”: The number of peptides detected by LC-MS/MS is a good benchmark to investigate the fraction in which the target protein is dominantly present. If the number of peptides of a certain protein identified in the nucleoid fraction is larger than that of the other fractions, the protein is thought to be abundant in the nucleoid [50]. For instance, in the log phase of *E. coli*, 7 Fis peptides were detected in the nucleoid fraction, and 2 in the envelope fractions, suggesting that Fis was more abundant in the nucleoid fraction. Here, we need to pay attention to the total number of peptides detected by the LC-MS/MS because more peptides should be detected if more sample is loaded for the LC-MS/MS [51]. In the case of the log phase of *E. coli*, 7148 peptides from 401 proteins were detected in the nucleoid fraction, and 13657 peptides from 334 proteins in the envelope fraction (Figure 2). Therefore, the relative abundances of Fis peptides in the log phase of *E. coli* were 9.8×10^−4^ (7/7148) and 1.5×10^−4^ (2/13657) in the nucleoid fraction and the top fraction, respectively, and the ratio was 6.7 (9.8×10^−4^/1.5×10^−4^). We arbitrarily selected proteins with a ratio higher than 3 as csNAPs.

**Figure 6:**
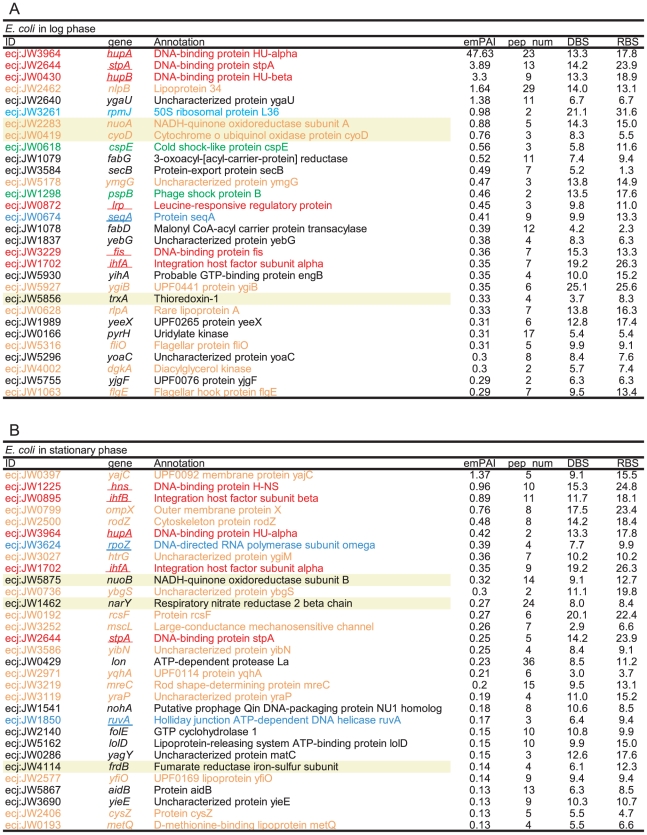
csNAPs of *E. coli.* The listed proteins were the top 30 csNAPs sorted by the emPAI values. (A) log phase and (B) stationary phase. The meanings of the colors, letters, and underlines are the same as those for Figure 4. The proteins with a yellow background are the oxidation-reduction enzymes. DBS and RBS represent the number of DNA-binding sites and RNA-binding sites per amino acid predicted by BindN [88], respectively. Values over 10 indicate high possibilities to bind to DNA and/or RNA (see materials and methods).

**Figure 7:**
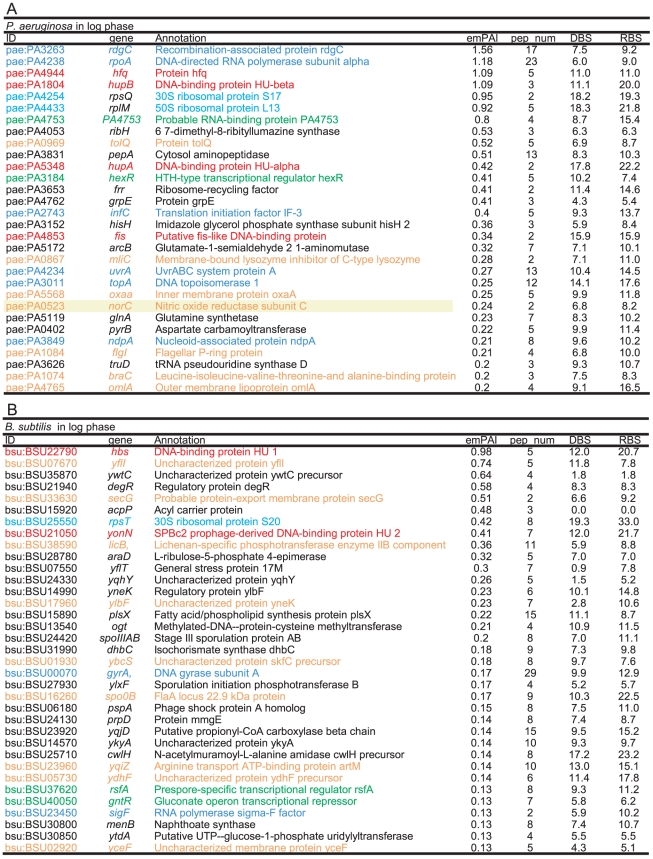
csNAPs of *P. aeruginosa* and *B. subtilis.* The listed proteins were the top 30 csNAPs sorted by the emPAI values. (A) log phase of *P. aeruginosa* and (B) log phase of *B. subtilis.* The meanings of the colors, letters, and underlines are the same as those for Figure 4. The proteins with a yellow background are the oxidation-reduction enzymes. DBS and RBS represent the number of DNA-binding sites and RNA-binding sites as described in Figure 6.

**Figure 8:**
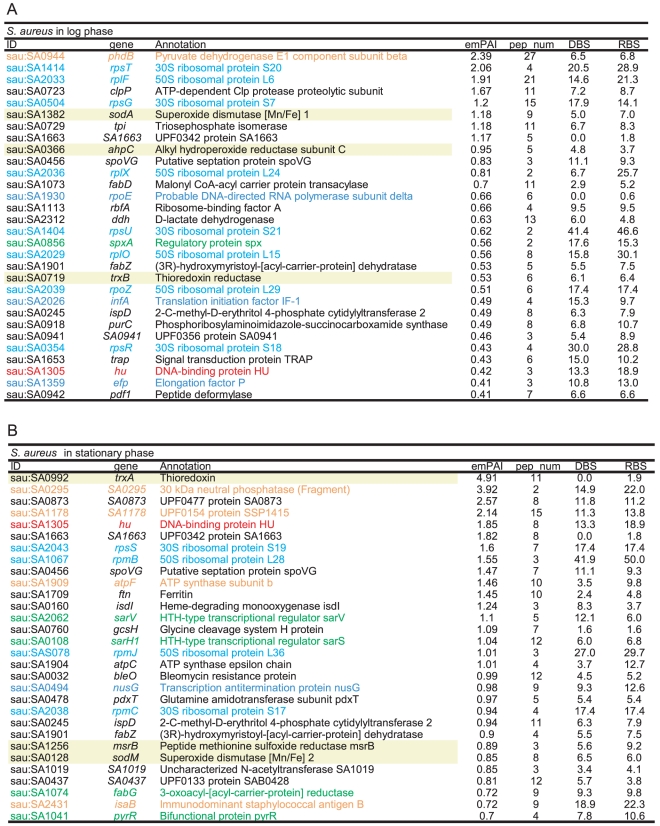
csNAPs of *S. aureus.* The listed proteins were the top 30 csNAPs sorted by the emPAI values. (A) log phase and (B) stationary phase. The meanings of the colors, letters, and underlines are the same as those for Figure 4. The proteins with a yellow background are the oxidation-reduction enzymes. DBS and RBS represent the number of DNA-binding sites and RNA-binding sites as described in Figure 6.

According to the above-mentioned criterion, 164, 66, 98, and 92 proteins were selected as csNAPs from the log phases of *E. coli*, *P. aeruginosa*, *B. subtilis*, and *S. aureus*, respectively (Figure 2, Figure 6, 7, 8, and Table S9, S10, S11, S12). From the stationary phase, 76 and 141 proteins were selected from *E. coli* and *S. aureus*, respectively (Figure 2, Figure 6, 7, 8, and Table S13-S14).

Various major envelope and cytosolic proteins were excluded in this operation. For instance, in the log phase of *E. coli*, all the porins (*ompA*, *ompC*, *ompF*, *ompN*, *ompT*, and *ompX*), most of the flagellar components (*fliC*, *fliF*, *fliL*, and *fliM* in both phases, but not *fliO* in the log phase), and many of the F_0_F_1_-ATPase subunits (*atpA*, *atpD*, *atpE*, *atpF*, and *atpG* in both phases, but not *atpB* and *atpH* in the log phase), chaperones (*groEL*, *groES*, *tig*, etc.), and metabolic enzymes (*tnaA*, *nuoC*, etc.) were excluded.

In contrast, the major NAPs, such as Hu (*hupA* and *hupB*), IHF (*himA* and *himB*), Fis (*fis*), and StpA (*stpA*), were judged to be csNAPs. H-NS (*hns*) and Hfq (*hfq*) were not included in the csNAPs in the log phase because they were also detected with high peptide numbers in the envelope and/or top fraction.

## Discussion

The csNAPs were selected as those proteins that were relatively abundant in the nucleoid fraction as compared with in the envelope and top fractions. It should be noted that the list of csNAPs is an incomplete one. Indeed, various proteins known to be included in the nucleoid (eg, ribosomal proteins, RNA polymerases, and some major NAPs such as HNS and Hfq) were eliminated from the csNAPs list. Nevertheless, it is reasonable to expect that the proteins selected as csNAPs are indeed involved in the functions of the bacterial nucleoid. Below, we discuss the csNAPs to gain insight into some of the characteristics of the bacterial nucleoid.

### Characteristics of csNAPs

When csNAPs of *E. coli* in the log phase are sorted according to the emPAI values that roughly represent the amount of proteins in the mixture [48], various DNA and/or RNA binding proteins, such as Hu (*hupA*, *hupB*) and StpA (*stpA*), are included in the top 10 *E. coli* csNAPs (Figure 6A). These proteins are global transcription/translation regulators that facilitate the response to various environmental changes [12]–[14]. In addition, other global regulators, such as CspE (*cspE*) [52], Lrp (*lrp*) [53], Fis (*fis*), and IHF (*ihfA*), were ranked in the top 20 *E. coli* csNAPs. In the stationary phase, global regulators including HNS (*hns*), IHF (*ihfA*, *ihfB*), Hu (*hupA*), and StpA (*stpA*) were involved in the top 20 (Figure 6B). Global regulators were also abundant in the csNAPs of *P. aeruginosa* (Figure 7A): The top 20 csNAPs of this bacterium included 5 global regulators, Hu (*hupA*, *hupB*), Hfq (*hfq*), Fis (*fis*), and HexR (*hexR*) [54], [55]. Thus, the csNAPs in the Gammaproteobacteira comprise a plentiful amount of global regulators. Other than Hu, such global regulator genes are not genetically conserved in *B. subtilis* and *S. aureus* (Firmicutes/Bacillales). Recently, staphylococcal accessory regulator A (SarA) and its homologues in *S. aureus* were proposed as global regulators [56]. Our csNAP list of *S. aureus* contains SarA (*sarA*) and its homologues SarR (*sarR*) and SarH1 (*sarH*) in both the log and stationary phases (Figure 8). In addition, the stationary-phase csNAPs include SarV (SA2062) and SarZ (SA2174).

Many functionally unknown proteins (y-genes) were also included with high emPAI values in the lists, especially in the *B. subtilis* list (Figure 7B). The prediction of DNA/RNA binding abilities showed that several y-genes had strong potential to bind DNA and/or RNA (Figure 7B, Figure S4, and Materials and Methods). There were 10 y-genes in the top 30 csNAPs of *B. subtilis*, and all of them encoded predicted DNA-binding (*yflI*, *yneK*, and *ydhF*) or RNA-binding (*yneK*, *ylbF*, and *ydhF*) proteins. It is possible that these genes encode novel global regulators.

An additional feature of csNAPs is the abundance of enzymes required for stress responses. For instance, in the log phase of *S. aureus*, the top 30 csNAPs included 3 reductase-like superoxide dismutases, *sodA*), alkyl hydroperoxide reductase (*ahpC*), and thioredoxine reductase (*trxA*), which have been reported to play important roles in coping with oxidative stress-responsive elements [57], [58]. In all the species, the various enzymes involved in the oxidative stress responses were selected as csNAPs. A NAP having such property has been found in the nucleoid of the plant plastid. Sulfite reductase (SiR), the enzyme that catalyzes the reduction of sulfite to sulfide in the sulfur assimilation pathway (see review in [59]), has been identified as a DNA-binding protein of the plastid nucleoid [60]. Recently, it has been proposed that SiR is also involved in oxidative stress resistance [61]. Although the present study only suggested the possibility of association/interaction of these enzymes with the nucleoid, it is possible that the bacterial genomic DNA is, in general, protected by these enzymes from reactive oxygen species, which we term the ‘armor hypothesis’ (see also Future Perspective in Discussion).

### Constitutive csNAPs through growth phases

**Figure 9:**
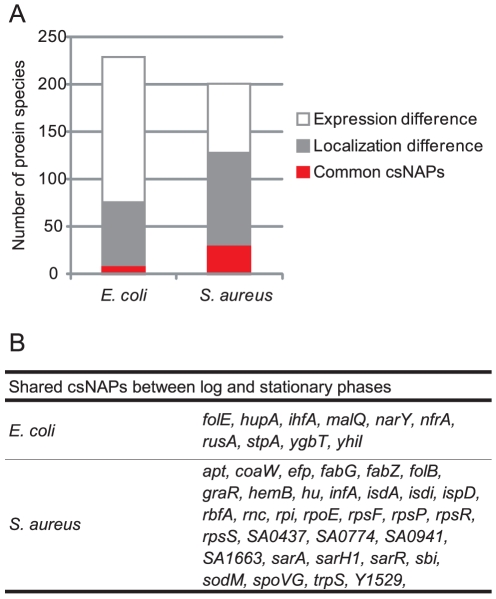
Common csNAPs between the log and stationary phases. (A) Classification of csNAPs Common csNAPs are csNAPs common to the log and stationary phases. Localization difference indicates the proteins which were not classified as csNAPs in either the log or the stationary phase but were detected in the envelope, and/or top fractions in the other phase. Expression difference represents the proteins which were classified as csNAPs in either the log or the stationary phase but not detected in any fractions in the other phase. (B) Common csNAPs between the log and stationary phases in *E. coli* and *S. aureus.*

The csNAPs in the log- and stationary-phase lists were different (Figure 9). In the list for *E. coli*, 4.3% of the csNAPs (10 out of 230 csNAPs [164 csNAPs in the log phase + 76 csNAPs in the stationary phase – 10 csNAPs present in both the log and stationary phases]) including Hu (*hupA*), StpA (*stpA*), and IHF (*ihfA*) were common between the log and stationary phases. In *S. aureus,* 15.9% of the csNAPs (32 out of 201 [92 in the log phase + 141 in the stationary phase – 32 present in both the log and stationary phases]) were common csNAPs throughout growth. One of the reasons for such a limited number of constitutive csNAPs could be the incomplete coverage of the proteins. Estimation of the real numbers of csNAPs on the basis of the coverage rates showed that the number of *E. coli* csNAPs may be 88 to 225 in the log phase and 62 to 85 in the stationary phase, while those of *S. aureus* may be 92 to 130 in the log phase and 127 to 169 in the stationary phase (Table S15). If all of the additional csNAPs overlap between the log and stationary phases, 34.8% of the csNAPs will be common in *E. coli* (80 out of 230 [225 in the log phase + 85 in the stationary phase – 80 present in both phases]). Accordingly, 45.3% of *S. aureus* csNAPs might be common (91 out of 201). Although it is unlikely that all the additional csNAPs overlapped, these data suggest that additional common csNAPs are constitutively present on the nucleoid.

Even considering the unidentified csNAPs, over 50% of csNAPs were expected not to be common. One reason would be the on/off of their expression. In *E. coli* and *S. aureus*, 66.5% (153 out of 230) and 36.3% (73 out of 201) csNAPs, respectively, exhibited log- or stationary-phase specific expression. Another reason seems to be the growth-dependent changes in localization of the csNAPs. For instance, *E. coli* HNS was detected both in the nucleoid and top fractions in the log phase (thereby not selected as a csNAP), but only in the nucleoid fraction in the stationary phase. *S. aureus* superoxide dismutase (SodA) appeared in the nucleoid fraction only in the log phase and was detected in all the fractions in the stationary phase. The percentage of csNAPs that exhibited such growth-dependent changes was 28.7% (66 out of 230) in *E. coli* and 47.8% (96 out of 201) in *S. aureus*.

Little is known about how NAPs change in the process of growing. It should be noted that the growth-dependent structural change of the nucleoid is induced in *E. coli*, but not in *S. aureus* [28], [33], [34], [62]. The increase in the amount of Dps and the decrease in Fis cause nucleoid condensation in the stationary phase in *E. coli*. On the other hand, the Dps orthologue, MrgA, is hardly expressed throughout the normal growth of *S. aureus*, and its nucleoid does not alter its apparent structure toward the stationary phase. The number of constitutive csNAPs that we could detect was smaller in *E. coli* than in *S. aureus*. This might imply the correlation between such structural change and the exchange of the NAP constituents.

Even under such growth-dependent changes, several global regulators and oxidation-reduction enzymes were constitutively present. In *E. coli*, Hu (*hupA*), IHF (*ihfA*), and StpA (*stpA*) were listed as global regulators, and nitrate reductase (*narY*) was listed as an oxidation-reduction enzyme. In *S. aureus,* the global regulators Hu (*hu*), SarA (*sarA*), SarR (*sarR*), and SarH (*sarH*) and the oxidation-reduction enzyme SodM (*sodM*) was constitutively present as csNAPs. These results suggest that global regulators and oxidation-reduction enzymes have important roles in the nucleoid regardless of their growths.

### csNAPs shared by *E. coli, P. aeruginosa, B. subtilis,* and *S. aureus*

**Figure 10:**
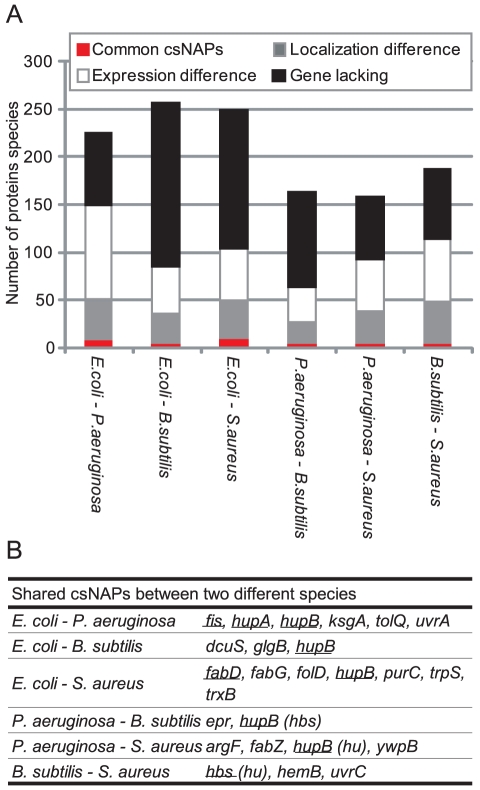
Common csNAPs in *E. coli*, *P. aeruginosa*, *B. subtilis,* and *S. aureus*. (A) Classification of csNAPs. Common csNAPs represent csNAPs common to 2 species. Localization difference means the proteins that were not classified as csNAPs in either 2 species but were detected in the envelope and/or top fractions in the other species. Expression difference indicates the proteins that were classified as csNAPs in either species but were not detected in any fractions in the other species. Gene lacking represents the proteins that were csNAPs in either species but for which the corresponding genes were not present in the other species' genomes. (B) csNAPs common to 2 species in the log phase. Underlined genes were ranked in the top 30 emPAI values in both compared species.

Comparison of csNAPs among species showed that Hu-β (coded by *hupB* in *E. coli, hupB* in *P. aeruginosa, hbs* in *B. subtilis,* and *hu* in *S. aureus*) was common to the 4 species. *E. coli* and *P. aeruginosa* share 5 additional csNAPs, whereas *B. subtilis* and *S. aureus* share 2 additional csNAPs (Figure 10). This low number of common csNAPs seems due both to ‘gene-level difference (the lack of the orthologous genes)' and to ‘protein-level difference (expression or localization difference).’ *E. coli* and *P. aeruginosa*, for example, did not share 33.9% of the csNAPs genes as orthologues (76 out of 224 [164 *E. coli* csNAPs + 66 *P. aeruginosa* csNAPs – 6 common csNAPs between these species]). Among the residual 148 csNAPs, whose genes are present in both species, 66.2% (98 out of 148) csNAPs were not detected in either *E. coli* or *P. aeruginosa*. While 30.4% (45 out of 148) of the csNAPs were detected in both species, they were not selected as csNAPs in either. *B. subtilis* and *S. aureus* showed a similar pattern. Although the degree of ‘gene-level difference’ increased in the distantly related species (41.8% to 66.9%), the ‘protein-level difference’ could still explain approximately half of the cross-species difference of the csNAPs (Figure 10A). As discussed in the previous section, it is possible that additional common csNAPs remain undetected (Table S15). However, the amount of those additional csNAPs in the cell should be small. Accordingly, the main csNAPs seem to differ among the species, suggesting that as each species evolved, it developed its own proteins associated with the nucleoid.

When the common csNAPs were sorted by emPAI values, Hu (*hupA*, *hupB*, *hbs*, *hu*), Fis (*fis*), and FabD (*fabD*) were included in the top 30 in certain pairs of species (Figure 10B). Hu gained high emPAI values in all the species. Given that it is crucial in various species [63]–[65], Hu should play a critical role in the bacterial kingdom. The abundance of Fis in *E. coli* and *P. aeruginosa* implies its importance in Gammaproteobacteria. It is interesting that FabD, also known as malonyl CoA-acyl carrier protein transacylase, was abundantly present in *E. coli* and *S. aureus*. In both species, *fabG*, which encodes 3-oxoacyl-[acyl-carrier-protein] reductase (FabG) and forms an operon with *fabD* [66], was also common. These genes are involved in fatty acid biosynthesis [67]. Between *P. aeruginosa* and *S. aureus*, FabZ, which is (3R)-hydroxymyristoyl-[acyl-carrier-protein] dehydratase and also involved in fatty acid synthesis, was a common csNAP. The prediction of their localization and DNA/RNA binding ability showed that these proteins are cytoplasmic proteins with less potential to bind DNA and RNA (Figure 6A and 6E). It might be possible that fatty acid synthesis occurs near the genomic DNA in those bacteria.

### NAPs not listed in the csNAPs: contaminant or genuine NAP?

Many proteins that have been reported to exist in the nucleoid were not included in the list of csNAPs. The involvement of the real NAPs in the top fraction might be due to disassociation from the NAPs during the purification procedure or might reflect their dynamic association with or disassociation from the nucleoid. Indeed, Hfq was reported to exist in both the cytoplasm and the nucleoid [68]. It might also be possible that high expression levels of NAPs lead to their higher accumulation in the cytoplasm owing to their saturation in the nucleoid.

Regarding the cell envelopes, the outer membranes of Gram-negative bacteria are resistant to nonionic detergents such as Brij-58 that was used in this study [5], [36]. It is likely that the outer membrane proteins detected in *E. coli* and *P. aeruginosa*, such as flagellin and porins, were contaminated owing to the insufficient solubilization of the outer membrane. The cell walls of Gram-positive bacteria are relatively thicker than those of Gram-negative bacteria. In particular, *S. aureus* possesses a thick cell wall that is resistant to lysozyme [69]. We used lysostaphin [70] to disrupt the cell wall, but it is possible that the cell wall fragments remained, in which case, cell-surface proteins such as immunodominant antigen A (coded by *isaA*) [71] and protein A (coded by *spa*) [72] in *S. aureus* would be the contaminants.

The inner membranes of *E. coli* are readily solubilized by Brij-58 [36], and indeed various inner membrane proteins, such as TonB [73], TolR [74], TatAB [75], and SecG [76], were detected only in the envelope fraction (Table S1). Nevertheless, some inner membrane proteins, such as the methyl-accepting chemotaxis proteins Tar and Tsr [77] were included in our nucleoid fractions (but not selected as csNAPs). These proteins may play some role in the nucleoid characteristics that require, for instance, interaction with the membrane. Similarly, Portalier and Worcel previously reported that several inner membrane proteins remained in the *E. coli* nucleoid even after treatment with sarkosyl and 1M NaCl [5]. In the present study, cytosolic portions of F_0_F_1_-ATPase (F_1_ β [*atpD*] and F_1_ γ [*atpG*]) were detected in the nucleoid and envelope fractions with high emPAI values under all the conditions tested. These remained in the nucleoid even after high salt treatment [45].

Recently, cell envelope-attached proteins, such as MreB, have been reported to interact with RNA polymerases and elongation factor EF-Tu [78], [79]. In *B. subtilis*, these proteins were indeed detected in both the nucleoid and envelope fractions. In *E. coli*, MreB (*mreB*) itself was not detected in the nucleoid fractions, but RpoZ (*rpoZ*), which is also a cytoskeleton protein and interacts with MreB [80], was detected in both the nucleoid and the envelop fractions. Thus, cytoskeleton proteins are also possible candidates for the linker of the nucleoid and cell envelope.

### Future Perspectives

Through the analyses of csNAPs in this study, we showed that global regulators, oxidation-reduction enzymes, and fatty acid synthases were enriched in the nucleoids. The distinct evolutionary origins of those proteins imply that bacteria have individually developed nucleoid-associated proteins in order to obtain similar characteristics.

While it was reasonable that various global regulators were in the nucleoid, it was surprising that oxidation-reduction enzymes and fatty acid synthase were enriched in the nucleoid because they have been believed to work in the cytosol and/or the envelope. The enrichment of oxidation-reduction enzymes facilitates our proposal of a new hypothesis - the armor hypothesis, which postulates the proteins in the nucleoid defuse the oxidative stress elements that challenge genomic DNA.

The presence of csNAPs involved in fatty acid synthesis implies certain relationships between the cellular membrane and the nucleoid. Phospholipids have been reported to regulate DNA replication via direct interaction with the replication initiator protein DnaA [81]–[83]. On the contrary, mutations in the replication machinery proteins such as DnaA, SeqA (negative modulator of initiation of replication), and Dam (DNA adenine methyltransferase) change the phospholipid constituents [84], [85], suggesting the presence of a bidirectional regulatory system to maintain the genomic DNA and cellular membrane. Fatty acid synthases in the nucleoid might also be involved in such a crosstalk system.

Although the current study only suggested the presence of the oxidation-reduction enzymes and fatty acid synthasesfattyf in the nucleoid, it would be fascinating in future studies to focus on nucleoid characteristics such as the ‘armor hypothesis’ and ‘nucleoid-membrane crosstalk.’

## Materials and Methods

### Bacterial strains and growth conditions

Glycerol stocks of *E. coli* K-12 W3110, *P. aeruginosa* PAO1, and *B. subtilis* 168 were inoculated in LB medium and cultured at 37°C with constant shaking (180 rpm; Bioshaker BR-15, TAITEC) for 18 h. Twenty-five microliters of the saturated culture was inoculated in 25 or 50 mL of fresh LB medium and cultured at 37°C with constant shaking (180 rpm) until the OD_600_ reached 0.7 (log phase) (Figure S1). The cell density was determined by measuring absorbance at 600 nm. Glycerol stocks of *S. aureus* N315 were inoculated in Brain Heart Infusion (BHI) medium (Difco, Detroit, MI, USA) and cultured at 37°C with constant shaking (180 rpm) for 18 h. Two hundred fifty microliters of the saturated culture was inoculated in 25 mL of fresh BHI and cultured at 37°C, with constant shaking, to an appropriate cell density (OD_600_ = 0.7). The cultures in the stationary phases were collected 12 to 14 h after the inoculations, and cells collected from 2 mL culture were used for the subsequent studies.

### Isolation of nucleoids

Cultures (25 mL for *E. coli*, *P. aeruginosa*, and *S. aureus* and 50 mL for *B. subtilis*) were centrifuged at 8000 g for 10 min at 4°C. Cell pellets were suspended in 0.5 mL ice-cold Buffer A (10 mM Tris-HCl [pH 8.2], 100 mM NaCl, and 20% sucrose) followed by the addition of 0.1 mL ice-cold Buffer B (100 mM Tris-HCl [pH 8.2], 50 mM EDTA, 0.6 mg/mL lysozyme [plus 100 µg/mL lysostaphin in the case of *S. aureus*]). The mixtures were incubated for the appropriate time at the appropriate temperature according to previous reports (*E. coli* [4] and *B. subtilis* [3]) or the preliminary experiments to understand the minimum requirements for the incubation conditions: in detail, 1 to 3 min on ice for the log phase of *E. coli*, 5 min at room temperature (RT) for the stationary phase of *E. coli*, 5 min on ice for the log phase of *P. aeruginosa*, 15 min on ice for the log phase of *B. subtilis*, and 15 min at RT for the log and stationary phases of *S. aureus*). Then, 0.5 mL ice-cold Buffer C (10 mM Tris-HCl [pH 8.2], 10 mM EDTA, 10 mM spermidine, 1% Brij-58, and 0.4% deoxycholate) was added, and the mixtures were incubated for the appropriate time (3–5 min at RT for the log phase of *E. coli*, 15 min at RT for the stationary phase of *E. coli*, 10 min at RT for *P. aeruginosa*, 20 min at RT for *B. subtilis*, and 30 min at RT for the log and stationary phases of *S. aureus*). The lysed cell suspensions were loaded onto linear sucrose density gradients containing 10 mM Tris-HCl (pH 8.2) and 100 mM NaCl (10%–60% for *E. coli*, *P. aeruginosa*, and *B. subtilis*, and 10%–30% for *S. aureus*) and centrifuged at 10,000 rpm with a Beckmann SW 40 Ti rotor (20 min for *E. coli*, 50 min for *P. aeruginosa* and *S. aureus*, and 40 min for *B. subtilis*). The DNA concentration in each fraction was quantified by DAPI fluorescence signals [4].

### Preparation of envelope fractions

Cellular envelopes were purified according to Zimmerman's method with several modifications [4]. The cultures (25 mL for *E. coli*, *P. aeruginosa*, and *S. aureus* and 50 mL for *B. subtilis*) were centrifuged at 8000 g for 10 min. Cell pellets were suspended in 0.5 mL ice-cold Buffer A followed by the addition of 0.1 mL ice-cold Buffer B (plus lysostaphin [25 µg/mL final concentration] in the case of *S. aureus*). The mixtures were incubated for the appropriate time (2 min at RT for *E. coli* and *P. aeruginosa* and 5 min at RT for *B. subtilis* and *S. aureus*). After adding PMSF (1 mM final concentration), the solutions were sonicated in ice water until they became clear. The debris was removed by centrifugation at 1200 g for 20 min at 4°C. The supernatants were collected, and 5 µg RNase, 10 U DNase, and MgCl_2_ (40 mM final) added. After 60-min incubation at 37°C, the envelope fractions were collected as pellets by centrifugation at 20,000 g for 60 min at 4°C.

### LC-MS/MS

Each lane of the Coomassie Brilliant Blue (CBB)-stained SDS-PAGE gels (8.5×6 cm) was cut into 10 sequential slices. Proteins in each gel slice were destained in 50% acetonitrile/50 mM ammonium bicarbonate (ABB), deoxidized by 10 mM DTT in 100 mM ABB, and alkylated by 55 mM iodoacetamide in 100 mM ABB. After being washed in 100 mM ABB and then 50% acetonitrile and 50 mM ABB, the gel slices were dried thoroughly in a MicroVac (Tomy Digital Biology, Tokyo, Japan). The proteins were then digested with 15 ng/µL trypsin (Trypsin Gold; [Promega, Madison, WI, USA]) in 50 mM ABB for 8 to 12 h at 37°C. Tryptic peptides were extracted by sonication in 50% acetonitrile/0.1% trifluoroacetic acid (TFA), and the supernatants collected. Again, the peptides were extracted by sonication in 75% acetonitrile/0.1% TFA and collected as supernatants. The samples were dried using the MicroVac and suspended in 2% acetonitrile/0.1% TFA. After being filtered by C-TIP (AMR Technology, Albany, NY, USA), the samples were analyzed by LC-MS/MS.

Reverse-phase nano-LC-MS/MS was performed using a Paradigm MS4 system (Michrom BioResources, Auburn, CA, USA) coupled to an LXQ (Thermo Scientific). The digested peptides were separated on an HPLC column (0.1×150 mm, 3 µm Magic C18AQ; Michrom BioResource) using a linear gradient of 6.4% to 41.6% acetonitrile in 0.1% formic acid for 20 min at a flow rate of 500 nL/min and detected by the ion trap in the 450–1800 m/z range following the supplier's recommendations. Mass spectra were acquired in the positive-ion mode with automated data-dependent MS/MS on the 3 most intense ions from the precursor MS scans.

Protein identification was performed using a Mascot Server (Matrix Science). Protein identifications were obtained by processing the experimental data against the SwissProt bacteria subset database (Release 57.4, June 16, 2009). The search parameters were as follows: trypsin was used as the cutting enzyme, mass tolerance for the monoisotopic peptide window was set to ±2 Da, the MS/MS tolerance window was set to ±1 Da, and 1 missed cleavage was allowed. Cysteine carbamidomethyl modification and oxidized methionine were chosen as the variable modifications. The criteria of positive identification were set as follows: identification of at least 2 peptides with more than 7 amino acids, and a significant threshold of *P*<0.05.

### Estimation of coverage rates

The coverage rates of proteins identified in each fraction were calculated by *in silico* simulation. We first created hypothetical protein sets (X-axis in Figure S2 B–D) and estimated the expected number of proteins identified by random sampling with a given number of the peptides (Y-axis in Figure S2 B–D). These plots make it possible to expect the actual number of the protein in the cognate samples. In detail, the steps of the procedure were as follows:

Step 1: Creation of hypothetical protein sets

We randomly collected 1 to 1500 protein sequences without overlaps from the genomic database of *E. coli*, *P. aeruginosa*, *B. subtilis*, or *S. aureus* [86]. The relative protein amounts (P_r_) in the hypothetical protein sets were given as follows: here we applied 3 different models: the constant model, the linear model, and the simplified canonical law (SCL) model (Figure S2A).


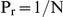
 constant model


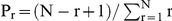
 linear model


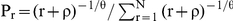
 SCL model,

where r is the rank of individual protein species sorted by their amounts, N is the number of the collected protein species (1–1500 in this study), and ρ and θ are the parameters to determine the individual protein amounts. In the constant model, the amounts of individual proteins were equal. In the linear model, the protein amounts linearly decreased. SCL was reported to fit well with the expression pattern in prokaryotic cells [47]. ρ and θ were set as 5.17 and 0.58, respectively, because Ramsden and Vohradsky showed that *E. coli* followed these values [47]. The ranks of the individual proteins were randomly determined. The number of the least protein was set as 1. Each of the collected protein sequences was replicated for the times according to the above statistical models. The summation of the number of replicated proteins was defined as the total amount of proteins. (We symbolize this value as M in the following steps.)

Step 2: Creation of theoretical peptide sets

The theoretical peptide sets that were theoretically produced by the digestion of trypsin were created from the hypothetical protein sets. Since the lengths of our detected peptides were between 6 and 45, we discarded the peptides with lengths out of this range.

Step 3: Estimation of the expected number of proteins identified in the hypothetical protein sets

Random sampling of the peptides from the theoretical peptide set was performed. Here, the number of the sampling cycles was set as the number of the experimentally identified peptides (eg, the log phase of the *E. coli* nucleoid as 7148). Then, the number of the selected protein species was counted (we counted only the proteins that were hit by more than 2 peptides according to our criteria for the protein identification). The amount of the selected proteins was set according to the number of the individual protein sequences determined in Step 1.

Step 4: Repetition of Steps 1 to 3 for calculation of the averages

We repeated Step 1 to 3 5 times and calculated the averages of the number of protein species (N_e_) and the amount of proteins (M_e_). Examples of the plots of N, M, N_e_, and M_e_ are shown in Figure S2 B to F.

Step 5: Estimation of the coverage rates

The coverage rates of protein species (R_N_) and amounts (R_M_) were calculated as follows:
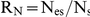


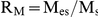



where N_es_ is the number of protein species identified in the real sample and N_s_ is the value of N when N_e_ gains N_es_. M_es_ and M_s_ are the values of M_e_ and M, respectively, when N_e_ gains N_es_. Examples of the R_N_ and R_M_ values are shown in Figure S2 G and H.

The number of peptides required for identifying all the potential proteins in the sample was estimated based on the above criterion of SCL model. We fixed the number of proteins in Step 1, and increased the numbers of peptides (with 1,000 intervals) to achieve the coverage rate of 1. The average of 5 trials is shown in Table S4. The above analyses were performed wholly by means of the Perl script.

### Comparative genomic analyses

The orthologue relationships were determined by best-best hit analyses using the FASTA package [87] on a desk-top computer. The pairs of proteins that showed a ‘best-best’ relation and a gain of less than 0.0001 of e-values were determined to be orthologous pairs. Total protein sequences identified in *E. coli* W3110, *P. aeruginosa* PAO1, *B. subtilis* 168, and *S. aureus* N315 were downloaded from KEGG on June 1, 2009 [86] and used for the best-best hit analyses.

### The prediction of DNA/RNA binding abilities

The DNA/RNA binding sites of the csNAPs were predicted by BindN [88]. The criterion for the search is ‘the predicted DNA/RNA binding residues with expected specificity equal to 90%.' We estimated the percentages of DNA/RNA binding residues in a protein and set 10% as the proteins having high DNA/RNA binding ability. This criterion was based on the following observations (Figure S4): The investigation of the number of DNA/RNA binding sites of Hu, IHF, HNS, StpA, Fis, and Hfq and of ribosomal proteins in *E. coli* showed that over 10% of the residues in each protein were predicted to be DNA/RNA binding sites. The distribution of the rates of the DNA/RNA binding sites of the csNAPs showed that 2 normal distributions appeared whose peaks were around 7.5% and 14% and that the gulf of the 2 distributions was around 10%. In contrast, the distribution of the protein detected only in the envelope and/or top fractions showed that only 1 normal distribution appeared whose peak was around 7.5%. These results support the notion that proteins whose predicted DNA/RNA binding sites are over 10% have high potential to bind to DNA/RNA.

### The number of potential csNAPs

The number of potential csNAPs (N_p_) was estimated based on the coverage rates of proteins and the number of subtracted proteins in the selection of csNAPs. N_p_ was calculated as follows:
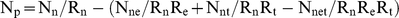



where N_n_ is the number of proteins identified in the nucleoid fractions, N_ne_ is the number of ‘overlapped proteins’ between the nucleoid and envelope fractions (‘overlapped proteins’ represent the proteins subtracted in the process of the csNAPs selection), and N_nt_ is the number of ‘overlapped proteins’ between the nucleoid and top fractions. N_net_ is the number of ‘overlapped proteins’ in all 3 fractions. R_n_, R_e_ and R_t_ represent the coverage rates of proteins in the nucleoid, envelope, and top fractions, respectively. The highest number of N_p_ was obtained when R_n_ was the lowest and R_e_ and R_t_ were the highest. The minimum N_p_ was obtained vice versa.

## Supporting Information

Figure S1 **Sampling points of bacterial cells.** The log phase cultures were collected at OD_600_ = 0.7, and the stationary phase cultures were collected 12 to 14 h after inoculation (arrows).
(TIF)

Figure S2 **The estimation of the coverage rates of the proteins in the samples.** (A) The probability distribution of protein amounts according to 3 different models: constant model (blue line), linear model (red line), and SCL model (green line). (B, C, D) The number of protein species expected to be identified in the hypothetical protein set with the constant model (B), the linear model (C), and the SCL model (D). The x axis represents the number of protein species in the hypothetical protein set, and the y axis, the expected number of protein species detected in the hypothetical protein sets. (E, F) The protein amounts expected to be identified in the hypothetical protein set with the SCL model (E), and the linear model (F). The x axis represents the number of protein species, and the y axis, the protein amounts expected to be identified. The solid line indicates the total amounts of proteins in hypothetical protein sets. (G, H) The coverage rates of protein species (G) and amounts (H) estimated with the SCL model. The x axis represents the number of protein species identified, and the y axis, the coverage rates of protein species (G) and protein amounts (H), respectively. The pink circle, square, and triangle indicate the points corresponding to the experimental results.
(EPS)

Figure S3 **Nucleoid isolation of** ***S. aureus*** **,** ***P. aeruginosa,*** **and** ***B. subtilis*** **.** The nucleoid isolations of the log phases of *S. aureus* (A, B), the stationary phase of *S. aureus* (C, D), the log phase of *P. aeruginosa* (E, F), and the log phase of *B. subtilis* (G, H). The spermidine nucleoids were fractionated by sucrose-gradient centrifugation with a 10%-to-30% (60%) gradient (A, C, E, G). The fractions containing genomic DNA were identified by DAPI fluorescence (B, D, F, H).
(TIF)

Figure S4 **The distribution of the percentages of DNA/RNA binding sites in proteins.** (A) The number of DNA/RNA binding sites in Hu, IHF, Fis, HNS, StpA, and Hfq in *E. coli*. Percent (%) represents the percentage of DNA (or RNA) binding amino acid in each protein. (B) The distributions of the percentages of the DNA/RNA binding sites of the csNAPs and the proteins that appeared only in the envelope and/or top fractions (env_top_specific) in *E. coli*.
(TIF)

Table S1 **Full list of the proteins identified in this study.**
(XLS)

Table S2 **Proteins identified in the nucleoid fraction of the log phase of** ***E. coli*** **.**
(XLS)

Table S3 **Proteins identified in the nucleoid fraction of the stationary phase of** ***E. coli*** **.**
(XLS)

Table S4 **Number of peptides required to cover all the potential proteins in samples.**
(XLS)

Table S5 **Proteins identified in the nucleoid fraction of the log phase of** ***P. aeruginosa*** **.**
(XLS)

Table S6 **Proteins identified in the nucleoid fraction of the log phase of** ***B. subtilis*** **.**
(XLS)

Table S7 **Proteins identified in the nucleoid fraction of the log phase of** ***S. aureus*** **.**
(XLS)

Table S8 **Proteins identified in the nucleoid fraction of the stationary phase of** ***S. aureus*** **.**
(XLS)

Table S9 **csNAPs of** ***E. coli*** **in the log phase.**
(XLS)

Table S10 **csNAPs of** ***E. coli*** **in the stationary phase.**
(XLS)

Table S11 **csNAPs of** ***P. aeruginosa*** **in the log phase.**
(XLS)

Table S12 **csNAPs of** ***B. subtilis*** **in the log phase.**
(XLS)

Table S13 **csNAPs of** ***S. aureus*** **in the log phase.**
(XLS)

Table S14 **csNAPs of** ***S. aureus*** **in the stationary phase.**
(XLS)

Table S15 **Potential number of csNAPs.**
(XLS)
