# Supplementary figures and images for: Proteomic Analyses of Nucleoid-Associated Proteins in Escherichia coli, Pseudomonas aeruginosa, Bacillus subtilis, and Staphylococcus aureus
Source: PLoS One. 2011 Apr 26;6(4):e19172. doi: 10.1371/journal.pone.0019172 (PMC3082553; doi:10.1371/journal.pone.0019172)

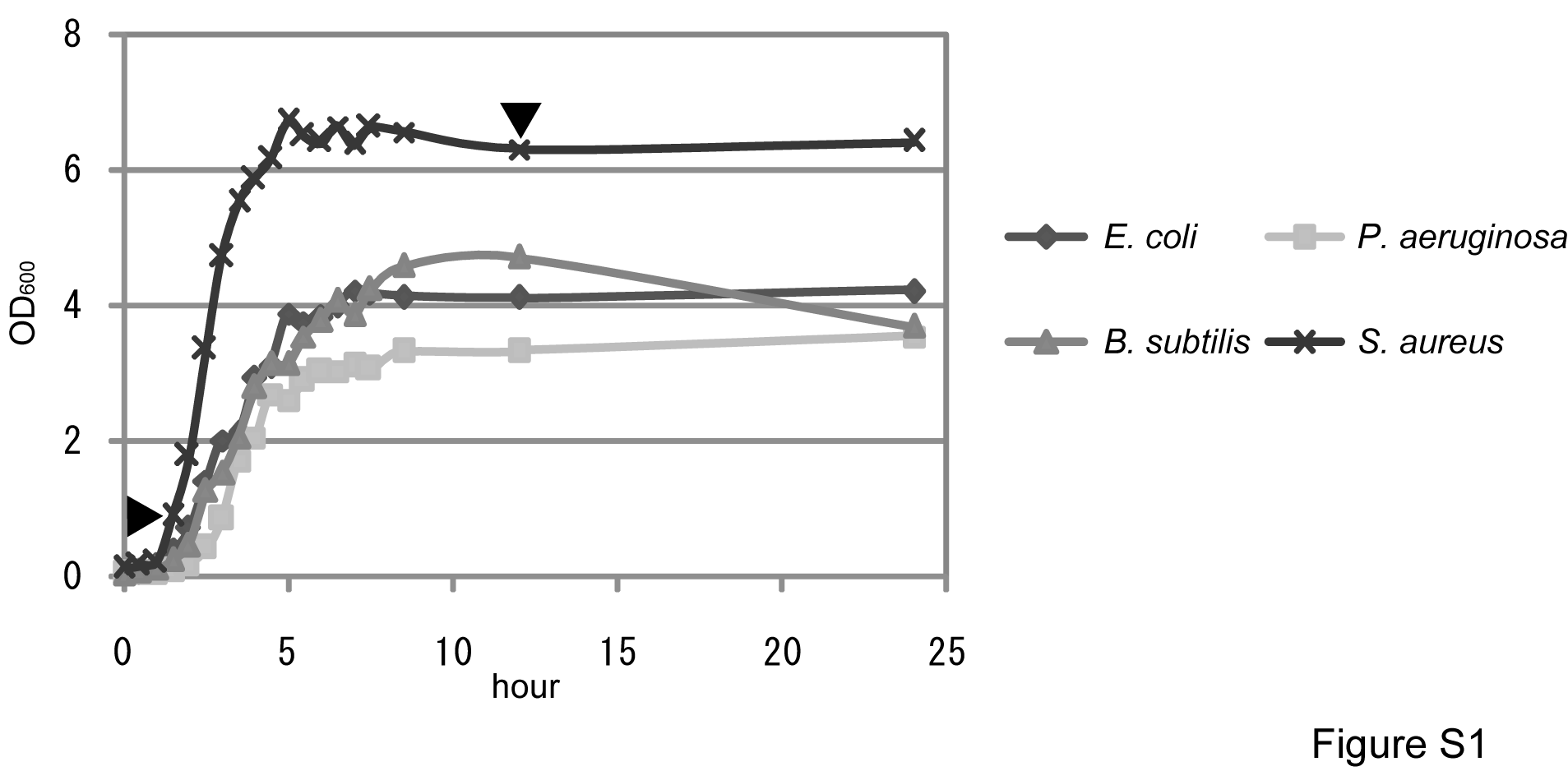

Supplement: Figure S1 — Sampling points of bacterial cells. The log phase cultures were collected at OD600 = 0.7, and the stationary phase cultures were collected 12 to 14 h after inoculation (arrows). (TIF) [file pone.0019172.s001.tif]

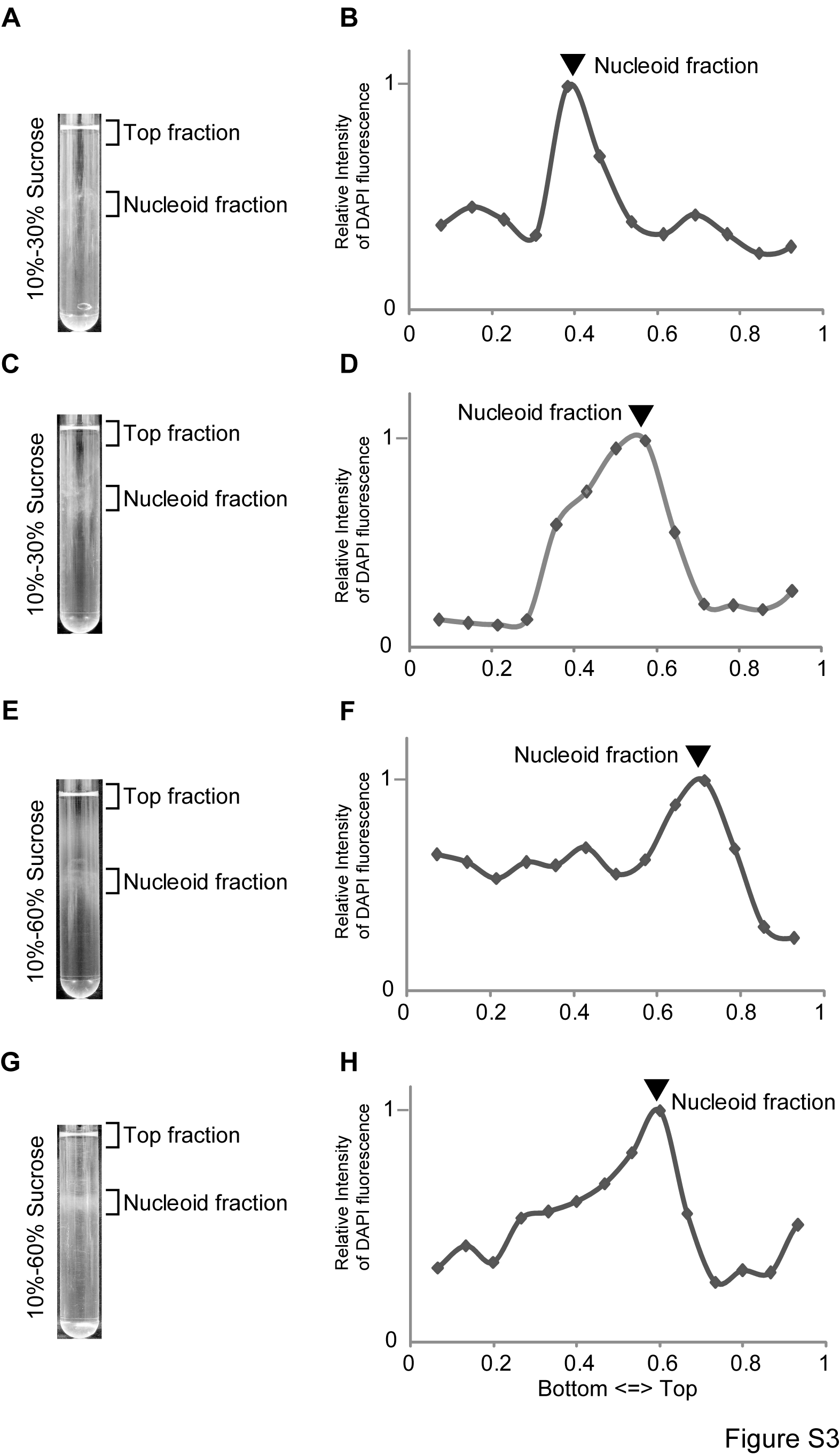

Supplement: Figure S3 — Nucleoid isolation of S. aureus , P. aeruginosa, and B. subtilis . The nucleoid isolations of the log phases of S. aureus (A, B), the stationary phase of S. aureus (C, D), the log phase of P. aeruginosa (E, F), and the log phase of B. subtilis (G, H). The spermidine nucleoids were fractionated by sucrose-gradient centrifugation with a 10%-to-30% (60%) gradient (A, C, E, G). The fractions containing genomic DNA were identified by DAPI fluorescence (B, D, F, H). (TIF) [file pone.0019172.s003.tif]

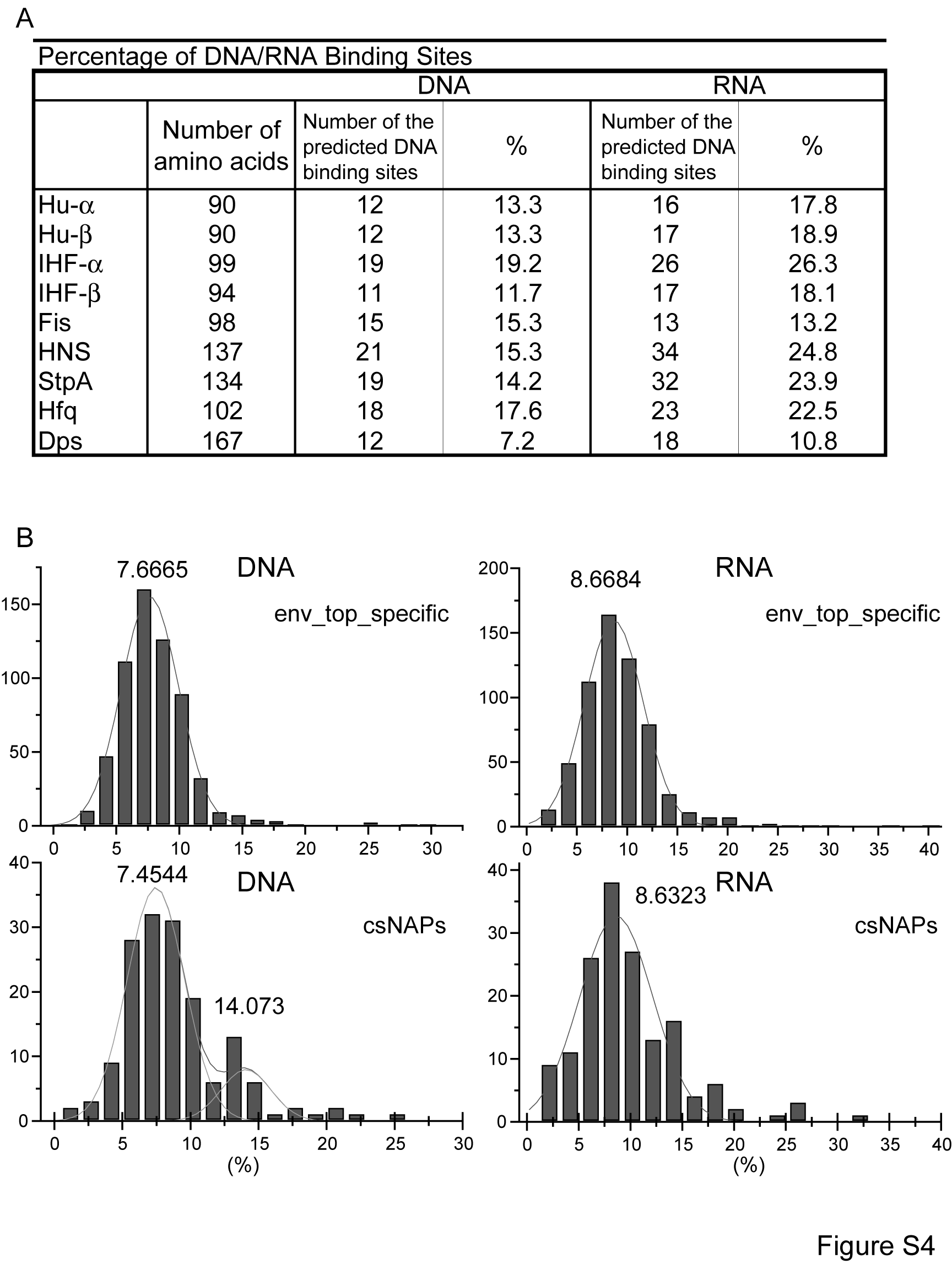

Supplement: Figure S4 — The distribution of the percentages of DNA/RNA binding sites in proteins. (A) The number of DNA/RNA binding sites in Hu, IHF, Fis, HNS, StpA, and Hfq in E. coli. Percent (%) represents the percentage of DNA (or RNA) binding amino acid in each protein. (B) The distributions of the percentages of the DNA/RNA binding sites of the csNAPs and the proteins that appeared only in the envelope and/or top fractions (env_top_specific) in E. coli. (TIF) [file pone.0019172.s004.tif]
